# Date (*Phoenix dactylifera*) Polyphenolics and Other Bioactive Compounds: A Traditional Islamic Remedy’s Potential in Prevention of Cell Damage, Cancer Therapeutics and Beyond

**DOI:** 10.3390/ijms161226210

**Published:** 2015-12-17

**Authors:** Bibi R. Yasin, Hassan A. N. El-Fawal, Shaker A. Mousa

**Affiliations:** 1 The Pharmaceutical Research Institute, Albany College of Pharmacy and Health Sciences, Rensselaer, NY 12144, USA; bibi.yasin@alumni.acphs.edu; 2 Neurotoxicology Laboratory, Albany College of Pharmacy and Health Sciences, Albany, NY 12208, USA; hassan.el-fawal@acphs.edu

**Keywords:** chemotherapy-induced adverse events, hepatotoxicity, mucositis, nephrotoxicity, peripheral neuropathy

## Abstract

This review analyzes current studies of the therapeutic effects of *Phoenix dactylifera*, or date palm fruit, on the physiologic system. Specifically, we sought to summarize the effects of its application in preventing cell damage, improving cancer therapeutics and reducing damage caused by conventional chemotherapy. *Phoenix dactylifera* exhibits potent anti-oxidative properties both *in vitro* and *in vivo*. This allows the fruit to prevent depletion of intrinsic protection from oxidative cell damage and assist these defense systems in reducing cell damage. Macroscopically, this mechanism may be relevant to the prevention of various adverse drug events common to chemotherapy including hepatotoxicity, nephrotoxicity, gastrotoxicity, and peripheral neuropathy. While such effects have only been studied in small animal systems, research suggests a potential application to more complex mammalian systems and perhaps a solution to some problems of chemotherapy in hepato-compromised and nephro-compromised patients.

## 1. Introduction

Cancer has long existed as a key task on the agenda of the United States Health Services. The “War on Cancer” was declared in 1971 with Richard Nixon’s signing of the National Cancer Act mandating the National Institutes of Health to “support research and the applications of the results of research to reduce the incidence, morbidity, and mortality from cancer”. However, in comparison to the reduction in mortality rate from heart disease, another top priority on the U.S. healthcare agenda, the decrease in cancer mortality rate has been modest [1]. It is therefore necessary to exhaust the medicinal potential of natural substances in addition to current chemotherapies to find a solution to such a large-scale problem. A variety of medications have been derived from nature-made sources, such as paclitaxel, derived from the Pacific Yew tree [2], and vincristine and vinblastine, derived from the Madagascar periwinkle [3]. However, these attempt to ameliorate problems retroactively when cancer is already established in a body. It is important to consider the potential of natural substances in prevention of illness and toxicities as well, especially those substances highlighted by religion and tradition that have been used medicinally throughout the ages, but whose medicinal properties have not yet been elucidated by scientific study. Thus, in this review, we seek to explore the potential of *Phoenix dactylifera*, commonly known as the date, in cancer prevention and therapeutics.

Madinah Al-Munawwarah, Saudi Arabia, and the adjoining areas are renowned as the home of the Ajwa variety of date. The inspiration for the pursuit of studying the medicinal properties of the date derives from Islamic Prophetic traditions mentioning dates, including some that point directly to its medicinal properties, and historic Islamic medical literature. The narrations point to both preventative and healing properties of the date. Ajwa dates themselves are highly prized as a result, carrying a price three times that of the next leading variety [4].

An examination of the available information on cancer incidence in countries where dates are widely consumed as a staple food source shows that Saudi Arabia, the world’s second largest producer of dates, rates “tumors” as fifth in their top 10 diseases according to the World Health Organization [5] and ranks far below the USA and the rest of the world for age-standardized rates of cancer incidence and mortality in males and females [6,7,8]. This can perhaps be linked to various aspects of the Saudi Arabian lifestyle, including the ways in which their diets vary from those of the Western world. In the U.S., cancer ranks second among the top 10 causes of death, according to the Centers for Disease Control [9] and has the sixth highest rate of cancer worldwide. The 2012 European Prospective Investigation into Cancer and Nutrition (EPIC) did a study of 477,312 subjects over 11 years to examine the effects of flavonoid (a type of polyphenol) and lignin intake on gastric cancer; women were a 60% majority of the subjects. A significant inverse relation between gastric cancer incidence and flavonoid and lignan intake in the female population was found [10]. Koreans have a high incidence of gastric cancer where dietary factors might play a key role; dietary flavonoids were shown to be inversely associated with gastric cancer risk in a case-control study with 334 cases and 334 age-matched controls [11]. Several other epidemiological studies suggested a potential for cancer prevention but further larger control studies are required [12]. However, no studies on polyphenol intake and cancer incidence have been undertaken in populations with diets that include dates, such as Middle Eastern or North African diets.

Studies on the effects of dates in the human body are lacking. For this review, available studies on the anti-oxidant effects and toxicity ameliorant properties of *Phoenix dactylifera* were sourced through a PubMed search using the keywords “*Phoenix dactylifera*”, “date fruit”, “date seeds”, and each in combination with the keywords “cancer”, “hepatotoxicity”, “nephrotoxicity”, “gastroprotective”, and “peripheral neuropathy”. Toxicity-related studies were limited to *in vivo* animal and human studies.

## 2. *Phoenix dactylifera* Constituents

The therapeutic effects of *Phoenix dactylifera* are attributed to its polyphenolic content [13]. Plant polyphenols are naturally occurring compounds found in fruit, vegetables, and in products such as fruit- and vegetable-derived sugars, juices, and oils. They are secondary metabolites in the plants they are produced from, serving as a defense system to ultraviolet (UV) light or pathogens [14].

The general chemical structure of polyphenols dictates their categorization into phenolic acids, flavonoids, stillbenes, ligans, and a multitude of derivatives and is a strong predictor of their rate of absorption and resulting serum levels [15]. Table 1 shows structural formulas of some of the varying polyphenols found in *Phoenix dactylifera*. All polyphenols arise from a common intermediate, known as phenylalanine, or a close precursor thereof called shikimic acid [14]. The products formed from these basic constituents are diverse, leading to a vast array of polyphenol protectants available to plants and humans alike.

Nature often finds a way to do what scientists fail to engineer into drug design—create synergy among substances, hence affecting polyphenolic metabolism and excretion. It is theorized that the health benefits of the synergy among polyphenol components in whole foods in our diet will far outweigh the benefits of any singular polyphenol [15]. Many researchers have thus opted to study whole fruit extracts to glean an understanding of the effects of the whole as opposed to its parts.

Plant polyphenols have been found to possess a range of effects: estrogenic and anti-estrogenic activity, anti-proliferative activity, induction of cell cycle arrest and apoptosis, prevention of oxidation, regulation of the host immune system, anti-inflammatory activity, modulation of effect of cytochrome P450 enzymes involved in activation of pro-carcinogens, upregulation of genes producing anti-oxidant enzymes, and the ability to change cellular signaling [15]. The challenge remains in standardizing these products for therapeutic and preventative consumption. 

Dates have been found to contain extremely high levels of phenolics, hypothesized to have formed due to exposure to extreme temperature and climate in comparison to other fruit [16]. The currently characterized polyphenols and average amounts in dried and fresh dates and date seeds are listed in Table 2 [17,18]. Nineteen different flavonoid glycosides of luteolin, quercetin, and apigenin have been found to exist in dates in methylated and sulfated forms. Analysis of mass spectral data has suggested that sulfates are linked to these flavonol glycosides as opposed to phenolic hydroxyls, making dates the only fruit or vegetable known to contain flavonoid sulfates. Anthocyanins have only been found to exist in fresh fruit, and carotenoids decrease rapidly as the fruit ripens [19].

The nutritional components of *Phoenix dactylifera* are plentiful (Table 3) [13,20]; Arab Bedouins historically subsisted on dates and camel milk [21]. The date’s capacity for retaining high nutrient value when dried made it a mainstay of desert region food staples. However, the processes that allow drying and thus preservation affect the bioavailability of the polyphenolic compounds. Upon drying, the date loses up to 30% of total carotenoids and 93% of anthocyanins. On the other hand, for the dried fruit, there is a statistically significant increase of 22%–153% in total phenolics per 100 g weight, differing by variety. Leading the way in this increase are phenolic acids, which have been found to have a statistically significant increase of 64%–107% in the dried fruit [17]. Total phenolic content in fresh dates is six times higher than in dried dates [16]. Storage of dried dates leads to oxidation of polyphenols, a potential explanation for this conflicting data.

**Table 1:** Chemical structures of some polyphenols found in *Phoenix dactylifera*.

**Phenolic Acids**	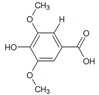	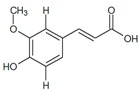
	Syringic acid	Ferulic acid
**Flavanoids**	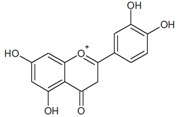	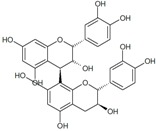
	Quercetin	Procyanidin
**Carotenoids**	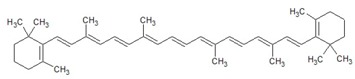	
	Beta carotene	
	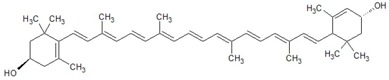	
	Lutein	

## 3. Anti-Oxidant Action of *Phoenix dactylifera*

A 2002 study showed aqueous date extracts to possess potent free radical scavenging activity [22]. Aqueous date extract of 0.8 mg/mL was shown to scavenge 50% of superoxide radicals formed by photoreduction of riboflavin and 100% of superoxide radicals at 1.5 mg/mL. In contrast, ascorbic acid did not scavenge superoxide radicals at all via this method up to concentrations of 1.5 mg/mL. A significant dose–response relationship in the inhibition of hydroxyl radical formation was observed via degradation of deoxyribose to thiobarbituric reactive substances (TBARS) by hydroxyl radicals, with concentrations of 2.2 mg/mL inhibiting 50% of hydroxyl radical formation, and 4.0 mg/mL inhibiting 100% of hydroxyl radical formation. For perspective, *Emblica oficinalis*, commonly known as “amla” in the Indian subcontinent, required an extract of 3.4 mg/mL to inhibit 50% of hydroxyl radical formation. Inhibition of lipid peroxidation was measured via ferrous/ascorbate system by the TBA method, and 50% inhibition was evidenced at 1.9 mg/mL and 100% inhibition at 4.0 mg/mL. In this realm, *Phoenix dactylifera* does not outstrip other anti-oxidant compounds, but has about half the capacity of amla, which inhibits 50% of lipid peroxidation at 1 mg/mL and 100% at 2 mg/mL. Inhibition of protein oxidation was also found to occur in a ferrous/ascorbate system by measurement of induced protein carbonyls. A concentration of 2.3 mg/mL reduced protein oxidation by 50%. Prior to this study, plant compounds were not found to inhibit protein oxidation *in vitro*, and this marks the first time such a phenomenon was observed and quantified. This study may have relevance to chemoprevention on a large scale because such minute quantities are easily consumed orally. However, its implications require an *in vivo* study to understand its full potential in a living system. Additionally, no mention of the specific species of *Phoenix dactylifera* was made, and it has been observed that polyphenolic content of dates as well as effect after consumption on serum oxidation levels in human models varies by species.

## 4. Potential Role of *Phoenix dactylifera* in Prevention of Oxidative Cell Damage

Current theory supports the idea that cancer is a result of oxidative damage to the cell by carcinogenic substances, causing cell cycle dysregulation. Reactive oxygen species production in the body can result in induction and maintenance of oncogenic cells via DNA mutation, a characteristic found to be overwhelmingly present in cancer cells in contrast to normal cells. Having established the anti-oxidant properties of the date fruit as comparable to or exceeding that of other fruits, and in smaller quantities, it is beneficial to consider its application in *ex vivo* and *in vivo* studies, particularly to gauge its merit in prevention of cell damage [23].

A 2009 study was done on the effects of Medjool and Halawi dates on serum glucose and lipid levels and on serum oxidative status [24]. Circumscribing the effects of the dates to polyphenolic content, researchers quantified total soluble phenolics in Hallawi and Medjool dates. Generally, the Hallawi variety was found to have a higher phenolic content of approximately 619 mg/kg in pyrogallol equivalents, and 1000 mg/kg in tannic acid equivalents, as compared to Medjool dates that contained approximately 517 mg/kg in pyrogallol equivalents and 763 mg/kg in tannic acid equivalents. The effects of oral consumption of both varieties of dates on serum oxidative status in 10 healthy, non-smoking subjects ranging in age from 20–40 years were quantified. It was found that 100 g of Hallawi dates consumed daily for four weeks significantly reduced basal serum oxidative status, AAPH-induced serum lipid peroxidation, and increased serum PON1 aryl esterase activity, an enzyme necessary to protect serum lipoproteins from oxidation. While these results show promise for future work, several caveats must be considered. Aside from the aforementioned demographics, no further demographic information was given for the subjects, and no clinical standard of “healthy” had been agreed upon by clinicians. In lieu of a control group, the subjects’ basal serum oxidative status was measured via TBARS assay prior to ingesting Medjool dates after the four weeks had elapsed, again after a four week washout period prior to ingesting Hallawi dates, and then again after ingesting Hallawi dates for the allotted four-week timeframe [24]. While this allows a contrast that is a true measurement of the subjects’ change in oxidative status, a separate control group that had undergone the same timing in measurements without the introduction of a treatment, or a null treatment, would have strengthened the study.

Two consecutive studies on date seed oil extract examined oxidative stress in an *ex vivo* model. Human melanocytes were harvested and incubated with date seed oil at a dose of 50 µg/mL, 24 h prior to exposure to 0.7 mM hydrogen peroxide, the concentration of which had been pre-determined according to its ability to reduce cell viability by 95%. The concentration of date seed had also been selected after pre-screening a range of doses to ensure that the selected strength was devoid of any significant negative effects on the cells. Date seed oil was found to significantly enhance cell viability after hydrogen peroxide exposure via the tryptan blue exclusion test. Depletion of glutathione peroxidase, superoxide dismutase, catalase, and lipid peroxidation were significantly reduced when measured with the TBARS test [25]. The study was later repeated with human keratinocytes, with a dose of 30 µg/mL selected after pre-screening a range of doses, and with oxidative stress induced by 0.5 mM hydrogen peroxide [26]; findings were similar to those of the former study. As both melanocytes and keratinocytes are implicated in the inflammatory processes of sunburn, these findings potentially have application to the prevention of skin cancer via treatment prior to sun exposure to prevent oxidative damage caused by UV radiation.

In an *in vitro* study on Salmonella tester strains TA 98 and TA 100, the potency of the extract was tested to gauge whether it was capable of inhibiting benzo(a)pyrene (BAP)-induced mutagenicity [22]. In both strains, date fruit extract was found to inhibit mutagenicity in a dose-dependent manner, measured by inhibition of His+ reverting colonies. Doses used ranged from 0.5 to 8 mg of fruit pulp, and they inhibited mutagenicity by 7% (0.5 mg) up to 80% (8 mg) in the TA 80 strain, and 3% (0.5 mg) up to 78% (8 mg) in the TA 100 strain. Of particular note is that BAP is a major product of cigarette combustion and a carcinogen implicated in the development of lung cancer [27]. Specifically, BAP is a pro-mutagen, requiring metabolic conversion for activation by cytochrome p450 enzymes in the liver to cause carcinogenic effects in the body. Taking this into account, this study too suggested that the date carries cancer preventative properties. The postulated mechanism of BAP activation involves “an oxenoid complex, which serves as an oxygen atom donor. This oxygen atom transfer process, mediated by the cytochrome P-450 system, may involve free radicals” [28], again supporting the case for the date’s potent anti-oxidant activity.

## 5. Potential of *Phoenix dactylifera* as a Chemotherapeutic Agent

The properties of *Phoenix dactylifera* cannot be demarcated merely by prevention, but extend beyond into the therapeutic realm of health care. One study isolated 1–6 branched and 1–3 branched β-glucans from date fruit extract to examine its effect on solid tumor growth. Sarcoma-180 tumor cells were subcutaneously implanted in mice, which were then treated with varying doses of date β-glucans. An inhibition ratio was calculated by comparing the average weight of the tumors of treated mice with untreated controls, and it was found that doses of 0.2 and 5 mg/kg resulted in significant inhibition of tumor growth. The intermediate dose of 1 mg/kg did not show significant inhibition, and both intra-peritoneal and intra-muscular administration are mentioned in separate parts of the paper [29]. This study is of particular interest because previous mass spectral data analysis of date fruit extract indicated that the flavonoid sulfates, unique to *Phoenix dactylifera*, are linked to the sugars of flavonol glycosides [19]. While the findings of this study require further research because the intermediate dose did not significantly inhibit tumor growth, a variety of β-glucans from differing plant sources, each with their own structural differences, have been shown to elicit a range of immune and anticancer responses. β-glucans have demonstrated inhibitory action on the growth of certain tumors in animal models, though clinical trials in cancer patients have not been performed. Many such studies have not used purified β-glucans, and were performed using crude extracts [30]. *Phoenix dactylifera*, thus, presents a β-glucan unique from the rest due to its flavonoid sulfates, allowing it to give a unique carbon nuclear magnetic resonance (NMR) signature that would allow direct isolation, purification, and use of this β-glucan for further chemotherapeutic study.

On the molecular level of cancer therapeutics, a study using date palm pit extract was performed to investigate anti-genotoxic effects on DNA damage induced by *N*-nitroso-*N*-methylurea (NMU) in mice [31]. The study evaluated both preventative and therapeutic potential of the extract, but the findings were heavily in favor of therapeutic treatment. Pre-treatment with date pit extract orally at a dosage of 25 mg/25 g daily after seven days showed no significant difference from control (not treated with NMU) in the number of abnormal metaphases present in the bone marrow cells. Animals in which DNA damage was induced prior to administration of date pit extract also showed no significant difference from control at 1, 2, and 7 days post-induction of DNA damage. The overall percentage of DNA fragmentation in all experimental groups was significantly elevated compared to control. The study would have benefitted from the application of a negative control that allowed examination of the effect of date-palm extract against a current standard of treatment in cancer treatment. The pre-treatment group at days 2 and 7 showed no significant difference *versus* control in numbers of micronucleated polychromatic erythrocytes, but again the group receiving date pit extract after DNA damage induction showed more immediate effects beginning at day 1, and continuing through day 7 after NMU application. These are the first steps into more elaborate studies on the effect of date palm pit’s therapeutic effects. In future studies, consideration should be given to use of quantitative morphometrics to identify and quantify the selected chromosomal aberrations to reduce the possibility of human error in quantification by eye via light microscope, as well as to allow quantification of a greater cell count closer to that used in the diagnosis of leukemia or other marrow-related cancers. Examination of other parameters required in diagnosis of marrow-related cancer, such as white and red blood cell count, is recommended to establish chemotherapeutic effects of date pit extract.

## 6. The Role of *Phoenix dactylifera* in Prevention of Chemotherapy-Induced Toxicities

### 6.1. Prevention of Hepatotoxicty

*Phoenix dactylifera*’s greatest potential may lie in application of its anti-oxidant effects to amelioration of adverse effects of standard chemotherapeutic treatments. One such area of research is amelioration of hepatotoxicity, commonly known to be caused by methotrexate, 6-mercaptopurine, 6-TG, azathiopurine, fluorodeoxyuridine, l-asparaginase, dactinomycin, gemtuzuab, radiation-induced liver disease, tamoxifen, cyproteron, and flutamide, among others [32]. Generation of reactive oxygen species (ROS), such as those generated by environmental chemicals and chemotherapy, are causative of the hepatic pathophysiologic changes that can eventually lead to liver cirrhosis, and hepatocellular carcinoma itself [33,34]. Accepted measures of clinical hepatic insufficiency in humans include an elevation in serum alanine aminotransferase (ALT), aspartate aminotransferase (AST), alkaline phosphatase, bilirubin, gamma-glutamyl transpeptidase (GGT), and hypoalbuminemia [35].

In a study of the protective effects of date palm fruit extract on dimethoate-induced oxidative stress in rat liver, date extract (4 mL/kg) was administered to experimental groups in combination with the hepatotoxicity-inducing agent dimethoate (20 mg/kg) [36]. To test the preventative effects of the fruit, an experimental group received an oral dose of date extract 30 min prior to administration of dimethoate for two months. This group was observed to have ALT and AST levels that did not differ significantly from the control group, which received 0.9% saline by oral gavage. This group’s ALT and AST levels were significantly lower than those of the negative control group, which had received dimethoate alone. To test the curative effects of the fruit, another experimental group received dimethoate for a month and then during the second month received date extract 30 min after dimethoate administration. This group had ALT and AST levels that were significantly elevated compared to control, but which were still significantly lower than a negative control group. Catalase, superoxide dismutase, and bilirubin levels were also not found to differ significantly from control, though a significant elevation of plasma alkaline phosphatase, GGT, and malondialdehyde (MDA) was found. Baseline tests on the rats were not conducted and would have further strengthened the findings by comparing the rats’ liver function before and after treatment. Characterization of the phenolic makeup of the extract used would also be useful to do in further studies.

Another study sought to elucidate the cytoprotective effects of date fruit on aflatoxin B1 (AFB1) toxicity [37]. AFB1 is well known as a potent inducer of hepatocarcinoma, and even low doses induce toxicity. Over the course of two weeks, date extract was administered to rats in tandem with AFB1 at 50 µg/kg. Plasma ALT, AST, creatinine, and urea levels were all found to be significantly higher than in the control group, yet were still significantly lower than in the group receiving AFB1 alone. Administration site of the extract and AFB1 in the date experimental group differed from that of other groups, which received the designated variable and AFB1 via intra-peritoneal administration, while the date experimental group received the dose via gastric tube. AFB1 requires the first-pass effect, or metabolic activation via the liver, to produce a metabolite capable of exerting biological effects; varying the route of administration of the toxin would certainly change the pharmacokinetics and pharmacodynamics of the toxin. This study deviated from the common date extract dose of 4 mg/mL, administering doses of 15 mg/mL to the rats; no particular rationale was noted. A standardization and repeat of this study is warranted to better understand the true impact of these findings.

One study focused specifically on the alleviation of ochratoxin-A (OTA) induced hepatotoxicity by Ajwa dates [38]. Rats receiving oral aqueous date extract of 1 g/kg/day two hours prior to administration of oral OTA at 289 µg/kg/day were found to have total bilirubin levels and serum ALT levels that were not significantly different from the control group. Of note in this study is the attention to standardization of the date extract dose to the equivalent Prophetic recommendation of seven dates per day, as well as administration of the date extract prior to OTA administration as a preventative measure. However, the results are skewed by the fact that the control group was administered sodium bicarbonate. As a result, one group receiving date extract without a concurrent hepatotoxic agent was shown to have bilirubin levels and ALT levels lower than the control group.

The therapeutic effect of date pit extract on carbon tetrachloride (CCl_4_)-induced hepatotoxicity was studied by Abdelaziz *et al.* [39]. The date pit extract was characterized and found to contain 38.8 mg Gallic acid equivalents of total phenolics and 87.86 mg of total flavonoids in rutin equivalents. Raw and roasted date pit extract at 1 g/kg was concurrently administered orally daily to rats in which hepatotoxicity was induced by intraperitoneal administration of 0.05 g CCl_4_ in olive oil twice a week. After the course of the four-week study, rats administered both roasted and raw date pit extracts were found to have significantly higher levels of albumin, and rats administered CCl_4_ alone had significantly decreased levels of ALT, AST, and alkaline phosphatase (ALP). These levels did not significantly differ from control rats that had been administered olive oil intraperitoneally twice weekly. Oxidative capacity of liver tissue in these rats was in part restored by both roasted and raw date pit extracts as indicated by the TBARS test. However, direct measurement of superoxide dismutase and glutathione-*s*-transferase showed that only roasted date pit extract produced a significant increase in these indicators of oxidative capacity in comparison to rats treated with CCl_4_ alone, and no significant difference from control, marking it as a restoration of normal oxidative capacity. Amounts of nitric oxide in both the raw and roasted date pit extract groups were significantly decreased from those in groups administered CCl_4_ alone, but also significantly increased from control, making this the only marker that was not restored to normal. These results confirm results of *Phoenix dactylifera*’s potent anti-oxidant ability although they are confounded by the use of olive oil as a vehicle and would have benefitted from the use of another nonpolar diluent lacking polyphenols as a vehicle for the hepatoxicity-inducing agent.

### 6.2. Prevention of Nephrotoxicity

The kidney is the other major metabolic organ that if compromised can lead to extreme patient hardship. Common nephrotoxic chemotherapeutic agents include diaziquone, cisplatin, ifosfamide, nitrosoureas, mitomycin C, plicamycin, antimetabolites, 5-azacytidine, high dose methotrexate, interferon, interleukin-2, gallium nitrate, cyclosporine, and tacrolimus [32]. Nephrotoxicity due to cisplatin is especially concerning because it is a widely reported complication of the agent’s use [40].

Cisplatin in particular presents an example of the paradox that appears to be posed by the administration of anti-oxidants prior to or concurrently with chemotherapeutic agents that generate ROS to lyse tumor cells, thus inducing toxicity. Endogenous thiols, such as those present in glutathione (GSH), were shown to completely prevent platination of DNA in peripheral blood mononuclear cells and ovarian cancer cells [41]. However, a dose of intravenous GSH 30- to 40-fold greater than the dose of cisplatin administered within 30 min of cisplatin administration has been found to be nephroprotective [42]. This is because GSH is the substrate for GGT localized to the cell surface. High levels of GSH inhibit GGT activity, thus inhibiting the metabolism of the cisplatin-GSH complex to a cisplatin-cysteinyl-glycine conjugate [40]. El Arem *et al.* [43] found that GSH levels in renal tissue in rats fed oral aqueous date extract for two months during concurrent administration of dichloroacetic acid did not differ significantly from control. This allows dates to be considered as an adjunctive agent that prevents nephrotoxicity via its actions on GSH, and perhaps would prevent withdrawal of patients from cisplatin therapy for whom it is an effective cancer agent, but has toxic side effects.

Several studies on the renoprotective effects of *Phoenix dactylifera* have been done and have shown great promise for future studies in human subjects. One was a two-month study in rats treated daily with date extract of 4 mL/kg, 30 min prior to renotoxicity induction with oral dimethoate (20 mg/kg), and a second experimental group that received dimethoate alone for a month and then began receiving the experimental treatment in the second month [44]. The study was designed to evaluate the preventative and therapeutic effects of date fruit extract on chemical-induced renotoxicity. Both pre-inducement and post-inducement experimental date groups had no significant difference from control in plasma creatinine, urea, uric acid, and renal superoxide dismutase. MDA levels, a major product of lipid peroxidation, were found to be significantly higher than control, but significantly decreased in comparison to treatment with dimethoate alone. Gluthathione peroxidase and catalase levels were shown to not significantly differ from control in the group that was pre-treated with date extract prior to dimethoate exposure, but the group that received date extract after a month of exposure to dimethoate significantly differed from control, but not from the dimethoate-alone group.

Another study in rats aimed to examine the effects of dichloroacetic acid on the kidney and whether date extract prevented nephrotoxicity. The total phenolic content of a 4 mL/kg aqueous date extract was characterized at 3.34 mg Gallic acid equivalents/100 g of fresh weight, and the entire polyphenolic composition was determined. Doses of 0.5 and 2 g/L of dichloroacetic acid (DCA) were selected, and experimental groups received the substance ad libitum via drinking water. Both doses of DCA were found to significantly increase plasma creatinine, urea, and MDA, and significantly reduced or depleted catalase (CAT), glutathione peroxidase (GPx), GSH, and superoxide dismutase (SOD) plasma levels. In contrast, the experimental groups receiving aqueous date extract daily showed no significant difference from control in any of the aforementioned markers, with the exception of SOD in the group receiving date extract and the higher dose of DCA (2 g/L). The doses of dichloroacetic acid were selected on the basis of a 0.2% concentration, which elicited a carcinogenic effect *in vivo*, and 0.05%, which is the lowest dose observed to cause liver tumors [43]. These results thus may have further implications for hepatocarcinoma not touched upon by the authors because they sought specifically to examine its nephrotoxic effects. While researchers hoped to design a study that would mimic human intake of DCA via drinking water, it would be useful for further studies to quantify the actual daily received dose. Baseline renal function of the rats is not given and is the only data point lacking in designating the findings of the study a restoration to normal for the animals.

Another study used both the fruit and pit extracts in alleviating the effects of a frequent kidney offender, gentamicin, in rats [45]. Parameters selected to measure the effects of the extracts were plasma creatinine and urea concentrations, the accepted measures of renal function, and kidney weight. The extracts were examined as preventative treatment and as ameliorative treatment. One group of rats received date fruit extract mixed with Purina chow as a 50% *w*/*w* mixture, and another group received date pit extract dissolved in distilled water in a 2:1 concentration as the only source of drinking fluid. Preventative treatment was tested via pre-treatment with each date component extract for 28 days, and administration of intramuscular (IM) gentamicin 80 mg/kg/day during the last six days of the timeframe. Ameliorative treatment was tested via concurrent administration of date component with 80 mg/kg/day gentamicin injections for six days. No significant differences in plasma creatinine and urea concentrations were found in any of the experimental groups in comparison to control. The date fruit and pit extracts were neither characterized nor quantified in this study and were mixed with Purina chow or drinking water without measurement of how much of the dose had been eaten. This type of study would have been more informative if the route of injection administration of gentamicin for ameliorative treatment was cited. In addition, olive oil, an alternate source of polyphenols, was used in the control group, and it could detract from the validity of the study’s findings. If polyphenolic content influenced the control group, a repeat study may potentially find an even larger difference between the experimental and null treatment.

### 6.3. Prevention of Gastrotoxicity

The effects of *Phoenix dactylifera* as a gastroprotectant were some of the initial studies performed on dates. The premise for these studies is gleaned from the Muslim Prophetic practice of eating dates to break the dawn to dusk fast during the holy month of Ramadan. Two consecutive studies sought to elucidate the meaning of this tradition via a rat model, one concerning gastric transit and the other the ameliorative effects of dates on ethanol-induced ulcers. The study on gastric transit has implications for cancer patients with chemotherapy-induced gastritis and for those patients on significant doses of pain medication because constipation due to the use of opioid narcotics is the only dose-limiting factor in treatment of severe cancer-related pain. The application of the study on dates’ effects on ulceration lies in chemotherapy-induced gastric mucositis, one of the most common adverse effects of treatment.

The study on gastrointestinal transit in rats examined several doses of aqueous and methanolic date extract, both dialyzed and undialyzed [46]. The primary measurement was the percentage of distance traveled by a charcoal-mixed meal in the intestine relative to the total length of the small intestine. Aqueous date flesh extract at doses of 0.02 and 0.04 mL/kg significantly reduced gastric transit time. These effects were dose-dependent. Aqueous dialyzed date flesh extract significantly increased gastric transit time again and was dose-dependent. Ethanolic date flesh extract (0.01, 0.02, and 0.04 mL/kg) significantly decreased gastric transit time, but was not dose-dependent. Aqueous date pit extract (0.01 mL/kg) significantly decreased gastric transit time, though the dose-dependence of this could not be proven because the same dose was applied to each group. Ethanol date pit extract at 0.02 and 0.04 mL/kg significantly decreased gastric transit time. It is apparent from these studies that the carbohydrate component of the date dictates its function in gastric transit time, either reducing it through its presence, or lengthening it via its absence.

The follow-up study reaped the benefit of these findings to explore implications for the alleviation of gastric ulceration [47]. The same range of aqueous and methanolic extracts of fruit and seeds at 4 mL/kg were used to pre-treat mice for a period of 14 days. On the last day of treatment, rats were given 80% ethanol to induce gastric ulceration and sacrificed after an hour. Aqueous date fruit extract (4 mL/kg), dialyzed aqueous date fruit extract (4 mL/kg), and ethanol date fruit extract all significantly reduced the number of ulcers, with ethanolic extract reduction not significantly different from positive control of lansoprazole (30 mg/kg). This finding suggests a natural alternative to the proton pump inhibitor turned to as a gold standard for the treatment of gastric ulcers. Gastrin (pg/mL plasma) and histamine (mg/g stomach weight) were also significantly reduced in all experimental groups, while mucin (µg/g stomach weight) was significantly decreased *vs.* negative control. No significant difference was seen between the experimental groups.

**Table 2:** Polyphenols in dates, and mean amounts if known.

		Fresh Fruit (mg per 100 g)	Dried fruit (mg per 100 g)		Seed (mg per 100 g)
**Phenolic acids**	**Hydroxybenzoic acids**				
	4-Hydroxybenzoic	0.16	0		
	Gallic	0.16	1.56		
	Protocatechuic	2.27	4.94		
	Syringic	2.45	6.06		
	Vanillic	1.76	4.13		
	p-Hydroxybenzoic				
	**Hydroxycinnaminic acids**				
	Caffeic	3.37	2.52	Protocatechuic	7.9
	Ferulic	9.62	11.83	Caffeoylshikimic (total)	28.3
	o-Coumaric	0.50	2.88		
	p-Coumaric	2.89	5.77		
**Flavonoids**	Apigenin			Apigenin derivative	0.5
	Quercetin			Quercetin derivative	3.4
	Luteolin			Proanthocyanidin dimer (total)	55.8
	Proanthocyanidines			Proanthocyanidin trimer (total)	61.3
	Anthocyanins			Epicatechin	18.8
**Lignans**		323.6 µg			
**Carotenoids**	B-carotene				
	Lutein				
	Lycopene				
	Violaxanthin				
	Flavoxanthin				
	Neoxanthin				
	Leukoxanthin				

**Table 3:** Date nutrition.

	**Calories (kcal/100 g)**	**Protein (g/100 g)**	**Fat (g/100 g)**
**Fresh fruit**	213	1.5	0.14
**Dried fruit**	314	2.14	0.38
**Major Phytoestrogens**		**Amount (µg/100 g)**	
Total		329.5	
Lariciresinol		116.9	
Pinoresinol		100.2	
Secoisolariciresinol		106.2	
Other		6.2	
**Minerals**		**Amount**	
Selenium, Copper, Potassium, Magnesium, Chromium		10%–19% of daily values	
Iodine		6 µg/100 g	
**Vitamins**		**Amount**	
A, B1, C		Trace	

### 6.4. Prevention of Peripheral Neuropathy

Peripheral neuropathy is another common adverse effect of chemotherapy. Taxanes such as docetaxel and paclitaxel, vinca alkaloids such as vincristine and vinblastine, oxaliplatin, thalidomide, and irinotecan are all commonly used agents that cause peripheral neuropathy. A study on streptozocin-induced diabetes in rats probed *Phoenix dactylifera*’s ability to prevent neuropathy [48]. Rats used in the study were screened for fasting blood glucose levels of 200 mg/dL, well over the standard for diagnosis in humans. Date fruit extract at 4 mL/kg was administered orally over a six-week timeframe post-confirmation of diabetic status. Measurements included rearing status, total distance moved, mobility duration, and grooming frequency using Ethnovision software, and motor nerve conduction velocity via direct stimulation at the right sciatic notch and ankle. No significant difference was found in rearing status, total distance moved, or mobility duration in the group receiving 4 mL/kg/day of date fruit extract orally, but a significant reduction from control was noted in grooming frequency and sciatic motor nerve conduction velocity. Baseline data on these animals would have further supported the results of the study, allowing comparison of the same animals before and after treatments.

## 7. Discussion

The World Health Organization has stated that the evidence supporting a decreased risk of developing cancer due to polyphenols, currently labeled ‘non-nutrient plant constituents’, is possible, but insufficient [49]. This puts the onus of responsibility on researchers to further explore the potential of these substances in cancer therapeutics. We reviewed *Phoenix dactylifera* as a plant polyphenol that may have vast potential for use in cancer therapeutics, given the available data. 

As evidenced in the majority of the studies, dates, fruit flesh and pits, do have the capacity to act as potent scavengers of reactive oxidative species. As such, they allow the body to sustain or recover its normal levels of endogenous enzymes such as catalases and peroxidases that protect the body at a cellular level when faced with toxicants. Most studies presented herein showed this via oral administration of date fruit or seed extract, the most common and advantageous administration route.

The anti-oxidant action of polyphenols and its application to cancer therapeutics appears to present a paradox because the majority of chemotherapeutic agents generate ROS as part of their therapeutic mechanism to lyse tumor cells. The role of ROS is not clear in cancer progression and as such the anticancer effects of dietary anti-oxidants are overcast by both promising and contradictory research findings [50,51,52]. It has been proposed that the failure of dietary anti-oxidants to consistently reduce carcinoma burden in clinical trials might due to the limitation of the dietary anti-oxidants to scavenge ROS at the tumor site [53]. Locally generated ROS at the tumor can activate various cancer-promoting activities including increased cell proliferation, decreased apoptosis, and increased metabolic adaptation [53]. On the other hand, the dietary polyphenol anti-oxidant epigallocatechin gallate (EGCG) demonstrated anticancer efficacy by itself in prostate cancer [54,55,56,57,58] and the anti-oxidant tempol analog, OT-404, demonstrated effective anticancer efficacy and enhancement of chemotherapeutic response [59].

Past studies indicated that oral administration of anti-oxidants was either ineffective or even induced the proliferation and metastasis of various cancers [51,52,60,61]. However, these studies utilized only the oral administration route for the selected anti-oxidants, subjecting them to first-pass metabolism and a variety of other *in vivo* modifications via the digestive system. In recent work, both *in vitro* and *in vivo* studies have demonstrated that anti-oxidants synthesized from natural sources decrease various tumor cell line viabilities, angiogenesis, and resistance to chemotherapy, leading to relapse [59]. Other anti-oxidants have been found that enhance chemosenstivity and reduce toxicity of concurrent chemotherapy [62]. *In vivo* studies with evidence in favor of anti-oxidants as adjuvants to chemotherapy have used alternatives to oral administration; specifically, the parenteral administration route for some anti-oxidants including glutathione, ascorbic acid, and OT-404 has proven more successful in reducing chemotherapy-related toxicity and adverse reactions, while also not interfering with the intended mechanism of such therapy [59]. Other studies have used oral administration routes for melatonin, ellagic acid, vitamin A, and α-tocopherol and have found significantly improved tumor response and survival rates while also reducing toxicities, and have been reviewed by Block *et al.* [63]. Because evidence exists to support both sides of the issue, anti-oxidant use with concurrent chemotherapy warrants further investigation into routes of administration and its application to different types of cancers.

### On the Horizon: Recommendation for Future Research and Studies

Measurements that are clinically meaningful in the human population must be conducted. For example, in measuring nephrotoxicity in humans, creatinine clearance and glomerular filtration rate are used in clinical practice as opposed to serum creatinine and serum urea. A gauge of the serum levels of polyphenols would also be useful in determining doses for use in higher organisms, as would use of current chemotherapeutic agents as opposed to agents known to induce similar toxicity. This last point is especially important because some mechanisms of toxicity of chemotherapeutics are not understood, thus requiring that ameliorative agents be put to test directly against the chemotherapeutics.

Studies using dates in humans will be held to a high standard. For high-impact studies to benefit medicinal science, the scientific method must always be used including adequate and appropriate controls, relevant biomarkers and clinical measurements, and procedure standardization. Characterization of the polyphenolic content and ratios of polyphenols in whole fruit extract is necessary as well, in the same manner medication doses are based on amounts of active pharmaceutical ingredients in a product.

Standardization does not imply a loss of the Islamic nature of the medicine. On the contrary, as shown by Abdu *et al.* [38], it is possible to standardize doses to the traditional recommendation of seven dates per day according to weight of the study subject. However, administration of the seven dates, or the equivalent in mice and other animals, by gastric gavage still does not standardize a study well enough to make future inferences concerning its results. Polyphenol content of such administrations must be homogenized and characterized to allow analysis of serum levels, pharmacokinetic and pharmacodynamics properties, and to understand the various fluctuations in fruit content caused by seasonal, regional, and environmental variation. This allows consideration of alternate delivery of active components of date extract, perhaps as sublingual, intravenous, or intraperitoneal formulations.

As with all substances introduced to medicine by the pharmaceutical industry, toxicity and bioavailability studies must be undertaken. Though the flesh and seeds have been consumed throughout the ages by humans and animals, controlled toxicity studies are necessary for date fruit; anything consumed beyond moderation has the potential to do more harm than good. Bioavailability studies are also key to exploring *Phoenix dactylifera*’s role in cancer therapeutics: how much a chemotherapeutic agent accumulates in blood and organs, and how much of a drug travels bound to plasma, and how much remains free. These parameters gauge effectiveness of medications and their potential to elicit a toxic response and are the standard for every clinical trial. It is apparent that *Phoenix dactylifera* exerts a wide range of effects on various organs of the body, and some of these applications will merit further investment of time and resources for pharmaceutical studies.
